# Integrated omics study of lipid droplets from *Plasmodiophora brassicae*

**DOI:** 10.1038/srep36965

**Published:** 2016-11-22

**Authors:** Kai Bi, Zhangchao He, Zhixiao Gao, Ying Zhao, Yanping Fu, Jiasen Cheng, Jiatao Xie, Daohong Jiang, Tao Chen

**Affiliations:** 1 State Key Laboratory of Agricultural Microbiology, Huazhong Agricultural University, Wuhan, 430070, Hubei Province, China; 2 The Provincial Key Lab of Plant Pathology of Hubei Province, College of Plant Science and Technology, Huazhong Agricultural University, Wuhan, 430070, Hubei Province, China

## Abstract

*Plasmodiophora brassicae* causes clubroot disease in cruciferous. In this report, lipid droplets were observed in the resting spores of *P. brassicae*. 295 lipid droplet-associated proteins were identified and categorized into nine groups. Transcriptome analysis of these proteins during three different zoosporic stages revealed differences in gene expression pattern. GO enrichment analysis revealed that these proteins associated with lipid droplets were mainly linked to biosynthesis and metabolism. GC-MS analysis revealed that lipid droplets contain seven types of free fatty acids: saturated fatty acids C16:0 and C18:0, and unsaturated fatty acids C18:1Δ9, C18:1Δ11, C18:2, C20:4 and C20:5. *P. brassicae* accumulated a large amount of triacylglycerols (TAGs). We systematically analyzed the putative proteins involved in TAG biosynthesis and its metabolic pathway. KEGG pathway analysis defined 3390 genes, including 167 genes involved in lipid metabolism. Transcriptome analysis revealed that 162 candidate enzymes involved in lipid metabolism were differential expressed. Our omics studies are the first to investigate the lipid droplet organelles in *P. brassicae*, providing a reference resource to study protist lipid droplets.

*Plasmodiophora brassicae* Woronin is an obligate biotrophic protist that infects cruciferous plants and is the causative agent of a disease commonly known as clubroot^1^,^2^,^3^. Clubroot is known to occur in more than 60 countries and results in an estimated 10–15% reduction of yields on a global scale^4^, which is characterized by the development of galls on infected rapeseed roots (Figure S1A–C). *P. brassicae* resting spores are often found in root cells and absorb plant nutrients to grow (Figure S1D,E). *P. brassicae* is a difficult study system, as it’s an uncultivable intracellular pathogen, and the pathogenicity of *P. brassicae* is poor understanding. *P. brassicae* is a member of eukaryotic supergroup Rhizaria^5^,^6^. The recently published genomes for *P. brassicae* show some typical biotrophic features of eukaryotic plant pathogens. Such as a small genome size with few repetitive elements, reduced number of CAZymes^7^,^8^, the incompleteness of some biosynthetic pathways suggest dependency on nutrient intake from the host, i.e. amino acids or lipids^7^,^8^.

*P. brassicae* has a complex and incompletely understood life cycle that consists of two main phases: root hair infection and cortical infection^9^. A primary zoospore is released from each resting spore. When the primary zoospore reaches the surface of a root hair, it builds a cyst and injects itself into the root hair. This stage is termed as the root hair infection stage. In root hairs the pathogen forms primary plasmodia, which develop into several single-nucleate secondary zoospores that are released into the soil. The root hair infection occurs in non-host plants as well as host plants. Primary zoospores or secondary zoospores can penetrate root cortex cell, induce cortical infection, in which the pathogen develops into secondary plasmodia. Proliferation of secondary plasmodia is associated with infected and neighboring uninfected cells undergo extreme cell division and expansion, leading to the formation of characteristic galls. After a number of nuclear divisions, the secondary plasmodia develop into multinuclear plasmodia and finally into resting spores, which are released into the surrounding soil when the gall disintegrates^10^.

Clubroot is one of the most difficult plant diseases to control because each large gall on an infected root contains millions of resting spores that can persist in the soil for up to 20 years^4^. In fungal dormancy carbohydrate and lipid composition of spores play a determinant role^11^. Plasmodiophorid spores contained a high abundance of lipid bodies^1^,^12^,^13^. The most abundant fatty acid in resting spores of *P. brassicae* is Arachidonic acid (ARA, 20:4)^14^. Transcriptional data suggest that fatty acids are synthesized in the plasmodia and degraded in resting spore^7^. Lipid droplet is a cellular organelle that stores neutral lipids [e.g., triacylglycerol (TAG) and cholesterol esters] as a source of energy and carbon. They serve as the energy reservoir for the entire organism^15^. Lipid droplets have been shown to be physiological important organelles in lipid metabolism rather than just storage organelles^16^. Numerous proteins have been shown by immunodetection or proteomic approaches to be associated with lipid droplets and to change under different physiological conditions^17^,^18^,^19^. Lipid droplets biogenesis and function have been studied in many organisms from archae, bacteria, plant to mammalian cells^20^. But until now lipid droplets proteins in Rhizaria including *P. brassicae* have not been studied. Here we report lipid with lipid droplets and their associated proteins will facilitate to improve our understanding of their functional roles for *P. brassicae*.

In addition to comprising cellular membranes, fatty acids are stored within cells as energy-rich TAGs in lipid droplets. In response to metabolic signals, mobilized fatty acids and other precursors derived from stored neutral lipids are used for a wide variety of functions within lipoproteins^16^. Fatty acids are essential metabolites used for energy production via β-oxidation. Because an excess of free fatty acids in the cytoplasm is toxic to cells, cells esterify fatty acids into neutral lipids and package them into lipid droplets^21^. The fatty acid compositions of lipid droplets and the mechanism underlying the fat metabolism in *P. brassicae* are still unknown.

In this study, we found that lipid droplets are present in the resting spores and second plasmodia of *P. brassicae* by transmission electron microscopy (TEM). Nile red staining showed that different zoosporic stages of *P. brassicae* contained lipid droplets. 295 lipid droplet-associated proteins were identified by proteomic study, and 7 types of fatty acid compositions were chemically characterized using GC-MS in lipid droplets. Genome integrated transcriptome, proteome of its lipid synthesis, storage and metabolism are detail described in *P. brassicae*.

## Results

### Nile red staining for detection of *P. brassicae*

**Figure 1:**
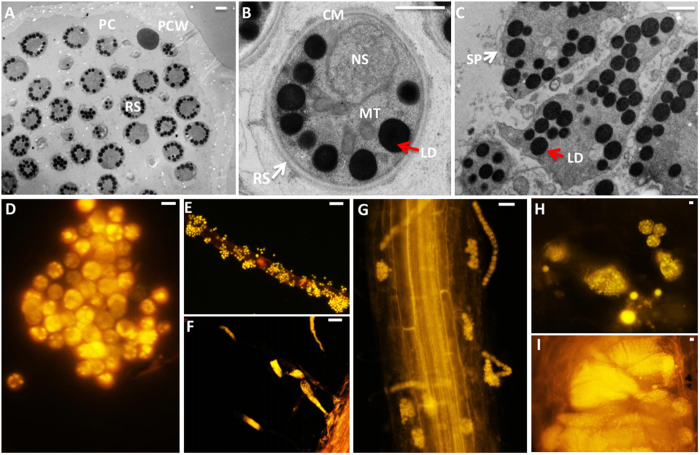
The observation of *B. rapa* galls infected with *P. brassicae*. (**A**) Transmission electron microscopy (TEM) of resting spores of *P. brassicae* in clubroots. (**B**) TEM of resting spores of *P. brassicae*. (**C**) TEM of secondary plasmodia of *P. brassicae*. RS = resting spores, white arrow; LD = lipid droplets, red arrow; PC = plant cell; PCW = plant cell wall; NS = nucleus; CM = cell membrane; MT = mitochondria. SP = second plasmodia, white arrow. Scale bar A-B = 1 μm. (**D**) Fluorescent Nile red staining for *P. brassicae* resting spores under a 100× oil objective. (**E**) Nile red staining for primary zoospores of *P. brassicae*. The primary zoospores are visualized after staining with nile red in Arabidopsis roots 2 days after infection, when they reached the surface of a root hair. They penetrated the cell wall and forms primary plasmodia in the root hairs (**F**). (**G**) Nile red staining for *P. brassicae* zoosporangia, which were visualized after staining with nile red in Arabidopsis roots 12 days after infection. H and I, Nile red staining of *P. brassicae* secondary plasmodia and resting spores in Arabidopsis roots at the root cortex cell. Arabidopsis roots infected with *P. brassicae* for 15 days were sectioned in the transverse plane (**H**). Arabidopsis roots infected with *P. brassicae* for 21 days (**I**). Scale bar = 5 μm.

TEM clearly revealed that the resting spores contain cell membrane, nucleus, mitochondria, lipid droplets and other organelles (Fig. 1A,B)^1^,^22^,^23^. Secondary plasmodia also had lipid droplets (Fig. 1C)^1^,^22^,^23^. The lipid droplets appeared to be round and heavily electron-dense in TEM images, and these results are consistent with those previously reports^22^,^23^. Nile red staining showed lipid droplets in *P. brassicae* resting spores (Fig. 1D,I), germinating spores (Fig. 1E), primary plasmodia (Fig. 1F), zoosporangia (Fig. 1G) and secondary plasmodia (Fig. 1H). These findings demonstrated that lipid droplets are present in *P. brassicae* throughout the root infection process at each observed stage.

### Lipid droplets were isolated and indentified from *P. brassicae* resting spores

**Figure 2:**
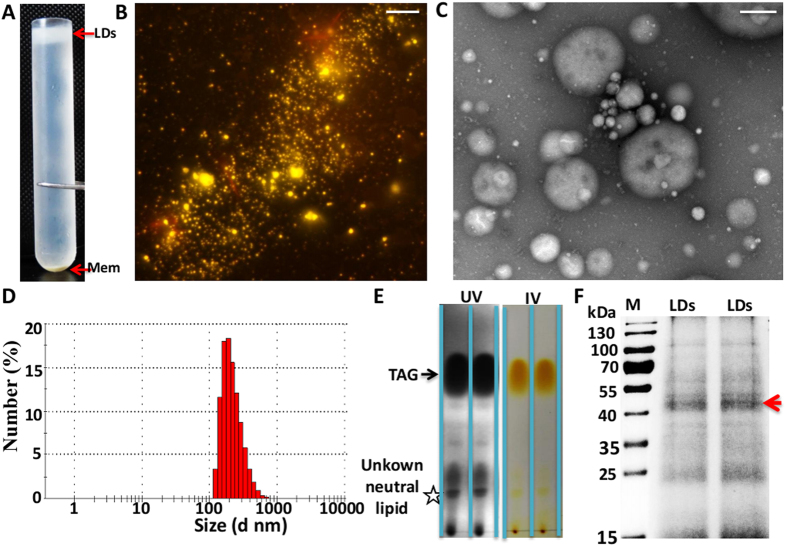
Isolation of lipid droplets from *P. brassicae* resting spores. (**A**) Spore suspensions are disrupted under high pressure and then centrifuged in SW40 tubes to remove nuclei, cell debris and unbroken cells. The LDs floated to the top of the gradient. (**B**) Lipid droplets stained with nile red, as viewed by fluorescence microscopy. Scale bar = 10 μm. (**C**) Isolated lipid droplets imaged by TEM after negative staining (scale bar = 0.5 μm). (**D**) TLC analysis of the total lipid extracted from isolated lipid droplets. (**E**) The size distribution of the purified lipid droplets. Values were generated using a Malvern Zetasizer Nanoseries (Malvern, England) equipped with a 633 nm laser. (**F**) Lipid droplets-associated proteins were extracted from *P. brassicae*, separated by 10% SDS-PAGE, and visualized by Coomassie blue staining. Arrows indicate the positions at which the gel was sliced.

To better characterize *P. brassicae* lipid droplets, we isolated the lipid droplets (Fig. 2A) and performed proteomic and free fatty acid analyses. Strong yellow-gold fluorescence signals were observed throughout the lipid droplets with nile red stained (Fig. 2B). TEM image with negative staining revealed that no membrane contamination in the isolated lipid droplets (Fig. 2C). The size of lipid droplets fits a spherical normal distribution ranging between 122 nm and 712 nm (Fig. 2D), similars to lipid droplets of the Gram-positive bacterium *Rhodococcus* sp. RHA1 (220 nm and 690 nm)^18^, and was confirmed to be similar to that previously visualized by TEM. The results of thin-layer chromatography (TLC) indicated that the lipid droplets were highly enriched in TAGs, and small amounts of unknown neutral lipids were also detected (Fig. 2E). Lipid droplet-associated proteins were separated on a 10% SDS-PAGE gel and subjected to coomassie brilliant blue staining (Fig. 2F) for proteimic analysis.

### Proteins associated with *P. brassicae* lipid droplets

**Figure 3:**
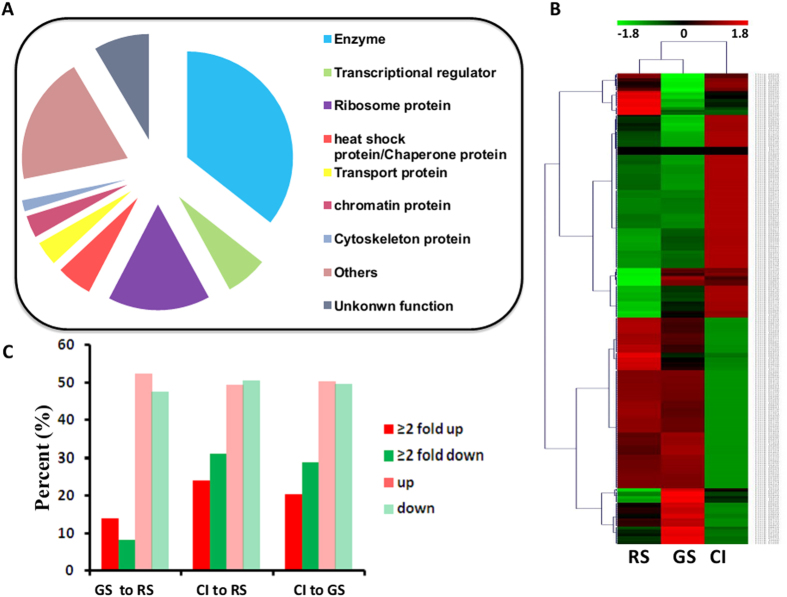
Functional and expressional analysis of lipid droplets-associated proteins. (**A**) 295 lipid droplet-associated proteins were identified in *P. brassicae* and categorized into nine groups based on genome searches and the KEGG and NCBI databases. (**B**) A heat map showing the expression of all 295 genes. (**C**) A histogram showing the percentage of lipid droplet–associated genes with dramatic differences in expression between the three life stages.

We used the whole gel sample (shown in Fig. 2F) and a fraction isolated from the main band in the gel (indicated by the arrow in Fig. 2F) for further proteomic studies. Among the 642 proteins found in the whole protein sample, 295 proteins were identified by at least two peptides (Tables S1 and S10). Of the 30 proteins detected in the isolated protein band (Table S2), 26 proteins were identified from the full gel sample, confirming that the proteomic data of lipid droplets are reliable. The 295 proteins identified in lipid droplets were categorized into nine groups, including 105 enzymes, 19 transcription and translation proteins, 46 ribosome proteins, 16 heat shock/chaperone proteins, 11 transport proteins, 10 chromatin proteins, 6 cytoskeleton proteins, 57 “other” proteins and 25 proteins of unknown function (Fig. 3A, Table S1). 14 enzymes were involved in lipid synthesis and degradation, including 3 transferases, 2 ligases, 7 dehydrogenases, 1 reductase and 1 synthase. 83 enzymes were involved in metabolic processes, while 28 were involved in biosynthetic processes ribosome, chaperone and histone proteins are abundant in the lipid droplets of *P. brassicae*, which had been identified in the lipid droplet proteomes of oleaginous bacterium^24^, *Caenorhabditis elegans*^25^, mammalian cells^26^, adult mice^19^ and *Drosophila* embryos^27^,^28^. Rab GTPase^29^, dynein, kinesin^30^, kinesin motor domain containing protein, actin^31^, 14-3-3 signaling proteins^17^ and heat shock protein 70^32^ were previously reported to play important roles in maintaining the functions of lipid droplets, and these proteins were identified in the lipid droplets proteome of *P. brassicae*. These data suggest that lipid droplets-associated proteins in *P. brassicae* were similar to those identified in other organisms.

### Function of lipid droplets-associated proteins deciphered by integrating transcriptomic and proteomic data

To investigate the proteins involved in lipid droplets, we sequenced and compared the whole-genome transcriptomes of purified mature resting spores (RS), germinating spores exposed to host root eluate (GS), and the cortex infection (CI, which contains the plasmodial stage of *P. brassicae*). The quality of our transcriptomes was confirmed by measuring the expression of 12 randomly selected genes involved in lipid metabolic pathways by qRT-PCR. Among these 12 genes, 10 genes (*PlasB_00083, PlasB_07509, PlasB_08640, PlasB_07041, PlasB_07695, PlasB_03260, PlasB_08647, PlasB_03885, PlasB_07155*, and *PlasB_09457*, Table S10) showed similar trends between transcriptomic and qRT-PCR analysis in these three stages. Whereas only 2 genes (*PlasB_07624* and *PlasB_03003*, Table S10) exhibited slight changes between transcriptomic and qRT-PCR analysis in these three stages (Figure S2 and Table S3). These qRT-PCR results confirmed that our transcriptomic data are reliable.

Transcriptome analysis of lipid droplets proteins showed that RS sample was mostly similar to that of the GS sample, but differed from that of the CI sample (Fig. 3B), suggesting that lipid droplets proteins exerted a greater effect at the CI sample than at the GS sample. Nearly half of the 295 identified proteins were differentially expressed (either up- or down-regulated) during the RS, GS and CI sample (Fig. 3C). The fraction of significantly up-regulated and down-regulated (|Fold change| ≥2 and adjusted p-value <0.05) genes between CI and RS as well as CI and GS was higher than that between GS and RS (Fig. 3C), suggesting that metabolic processes were accelerated during the CI sample. Furthermore, 1 lipase, 6 heat shock proteins, elongation factor 2, citrate synthase and ATP-dependent DNA/RNA helicase were significantly up-regulated, whereas 19 ribosomal proteins, Rab GTPase, isocitrate lyase and ubiquitin-protein ligase 1 were significantly down-regulated in CI compared with RS. We conducted gene ontology (GO) enrichment analysis for proteins associated with protist lipid droplets and found 197 proteins in the main cluster of 295 proteins. Of these, 79 proteins were involved in biological processes, and 154 proteins were involved in metabolic processes (Tables S4 and S5). The enrichment (78%) of proteins involved in metabolism is higher than that previously reported for the lipid droplets of *C. elegans* (41%)^33^. The abundance of proteins detected in *P. brassicae* lipid droplets can speculate that they are involved in not only lipid metabolism, but also other multiple important cellular functions.

Twenty-five years ago, lipid droplets were simply considered as inert cellular inclusions for the storage of neutral lipids, while there were hints from proteomics studies that droplets may interact with other structures to share lipids and proteins in the past decade^34^. We classified *P. brassicae* lipid droplets-associated proteins into 8 groups by predicted function or localization (Table S6)^27^,^34^. Our data showed that *P. brassicae* lipid droplets-associated proteins might play important roles in the interaction between the lipid droplets and endoplasmic reticulum, mitochondria, peroxisome, nucleus, vesicle, cytoplasm, membrane and ribosome, respectively (Table S6).

### Free fatty acid composition of lipid droplets in *P. brassicae*

**Figure 4:**
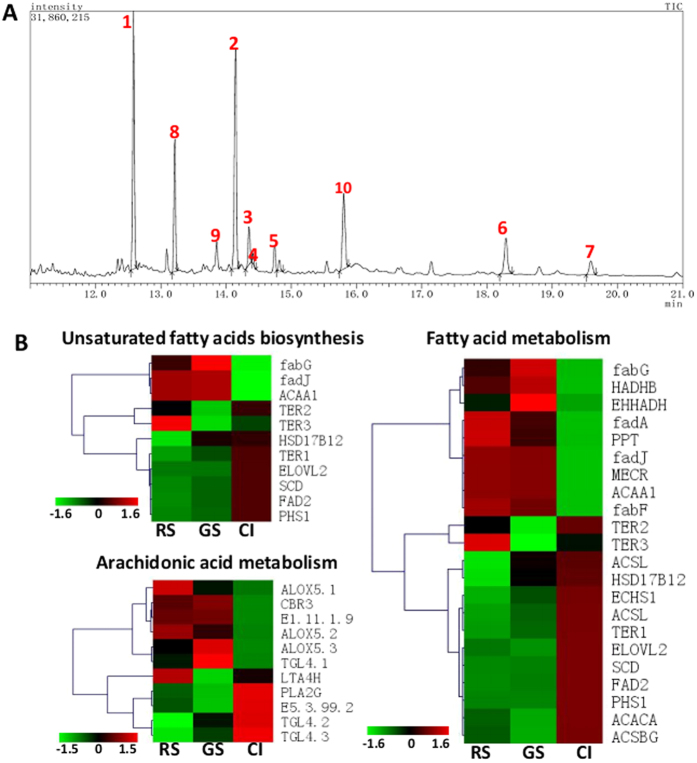
GC-MS analysis of lipid droplet free fatty acid content and expression heat maps of genes associated with unsaturated fatty acid biosynthesis, arachidonic acid metabolism, and fatty acid metabolism in *P. brassicae*. (**A**) GC-MS analysis of the free fatty acid methyl ester composition of lipid droplets. Note: 1. C16:0; 2. C18:0; 3. C18:1Δ9; 4. C18:1Δ11; 5. C18:2Δ9, 12; 6. C20:4Δ5, 8, 11, 14; 7. C20:5Δ5, 8, 11, 14, 17; 8. 2, 4-bis (1,1-dimethylethyl)-phenol; 9. Benzenepropanoic acid-methyl ester; and 10. C_32_H_66._ (**B**) Gene expression of genes in KEGG categories involved in unsaturated fatty acid biosynthesis, arachidonic acid metabolism and fatty acid metabolism. A colored bar indicating the normalized reads per LOG2 (RPKM) accompanies the expression profile.

Free fatty acids are formed by the hydrolysis of phospholipids and neutral lipids in neutral and acidic lipase through β-oxidation to produce energy. The free fatty acid content of the lipid droplets was determined using gas chromatography-mass spectrometry (GC-MS) to analyze fatty acid methyl esters. The peak intensities and characteristic ions of the 7 fatty acid methyl esters are shown in Table 1, and the GC-MS of each fatty acid methyl ester is presented in Fig. 4A. The saturated fatty acids are C16:0 and C18:0, the unsaturated fatty acids are C18:1Δ9, C18:1Δ11, C18:2, C20:4 and C20:5, and the proportion of saturated fatty acids and unsaturated fatty acids was 76.6: 23.4. It is worth mentioning that approximately half of the unsaturated fatty acids were C20:4 (arachidonic acid, ARA), which accounted for 10% of the total free fatty acid content. C20:5 (eicosapentaenoic acid, EPA) accounted for 4.2% of the total free fatty acid content. These results indicated that lipid droplets are rich of ARA and EPA.

Transcription data showed that genome KEGG pathway categories belonging to unsaturated fatty acid biosynthesis, arachidonic acid metabolism and fatty acid metabolism were present in all samples. Ketoacyl-acyl carrier protein reductases (FabG) are highly conserved and ubiquitously expressed enzymes of the bacterial type II fatty acid synthesis pathway; down-regulation was observed in the CI sample. FAD2 is the main enzyme responsible for unsaturated lipid synthesis, converting oleic acid (18:1) to linoleic acid (18:2); up-regulation was observed in the CI sample (Fig. 4B). Lipoxygenase enzymes catalyze the conversion of arachidonic acid into biologically active lipid mediators in the form of three 5-lipoxygenase enzymes (ALOX5.1, ALOX5.2 and ALOX5.3); up-regulation was observed in the RS and GS stages. Arachidonic acid metabolism and fatty acid metabolism occurred in all stages (Fig. 4B and Table S10).

### Enzymes involved in TAG biosynthesis and metabolism

**Figure 5:**
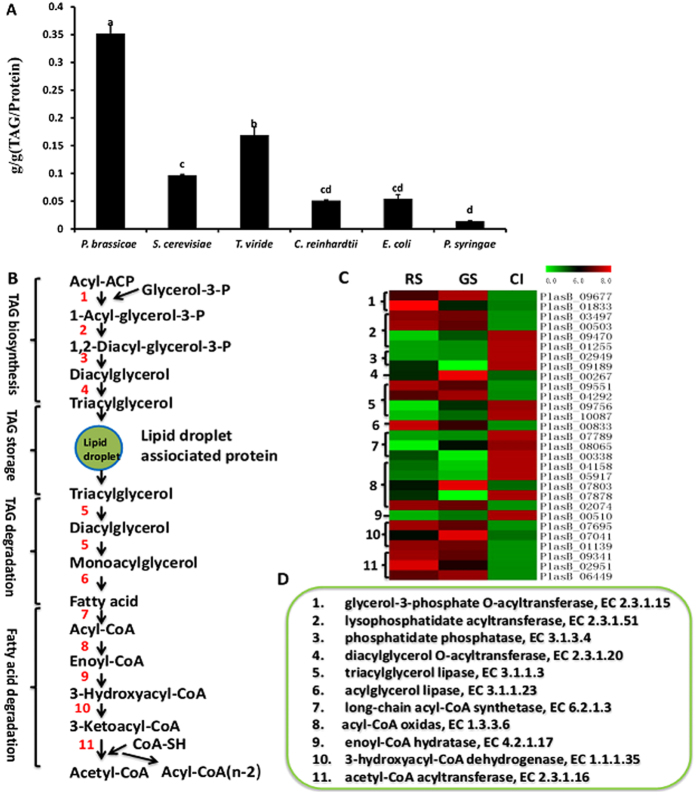
Measurement of TAG contents and an expression analysis of gene families involved in TAG biosynthesis and degradation. (**A**) Measurements of the TAG contents of multiple species. TAG content was assessed using a tissue glyceride assay kit from APPLYGEN. Protein content was assessed using a Total Protein Assay Kit from Thermo Scientific. Data are means from three independent experiments; error bars show the SD. p < 0.01 (spss). *Plasmodiophora brassicae: P. brassicae, Saccharomyces cerevisiae: S. cerevisiae, Trichoderma viride: T. viride, Chlamydomonas reinhardtii: C. reinhardtii, Escherichia coli: E. coli*, and *Pseudomonas syringae: P. syringae*. (**B**) TAG biosynthesis, storage and degradation pathways are divided into 4 biochemical stages and 11 reactions. (**C**) A heatmap of the enzymes involved in each reaction. (**D**) EC numbers of enzymes involved in (**B**).

The most important compounds of lipids for energy storage in mammals are TAGs. *P. brassicae* accumulates the largest amounts of TAG from multiple species we examined, such as *Saccharomyces cerevisiae, Trichoderma viride, Chlamydomonas reinhardtii, Escherichia coli* and *Pseudomonas syringae* (Fig. 5A). KEGG pathway analyzed the potential proteins involved in the TAG biosynthesis and metabolic pathway. The pathway consists of 11 reactions, which are further classified into 4 stages: TAG biosynthesis, TAG storage, TAG degradation and fatty acid degradation (Fig. 5B,D). Among these reactions, 29 enzymes related genes were differentially expressed at all developmental stages in *P. brassicae* (Fig. 5C and Table S10). By using BLAST2GO to analyze the potential proteins, 26 proteins were shown to be homologous to lipases (15 lipases and 11 phospholipases), and *PlasB_09551, PlasB_04292, PlasB_09756* and *PlasB_10087* were shown to be homologous to triacylglycerol lipases; the expression of *PlasB_09756* and *PlasB_10087* was significantly elevated in CI compared with in RS (≥2-fold change), while *PlasB_09551* and *PlasB_04292* were significantly down-regulated in CI compared with in RS (≤−2-fold change) (Figure S3A and Table S10). 44 proteins were predicted to be homologous to esterases (10 esterases, 21 phosphatase and 13 thioesterases). These proteins can be classified into some subclusters with similar expression patterns (Figure S3B, Table S10). These results suggest that those differentially expressed lipases/esterases may be involved in TAG synthesis and degradation.

### Genes and pathways involved in lipid metabolism in *P. brassicae*

**Figure 6:**
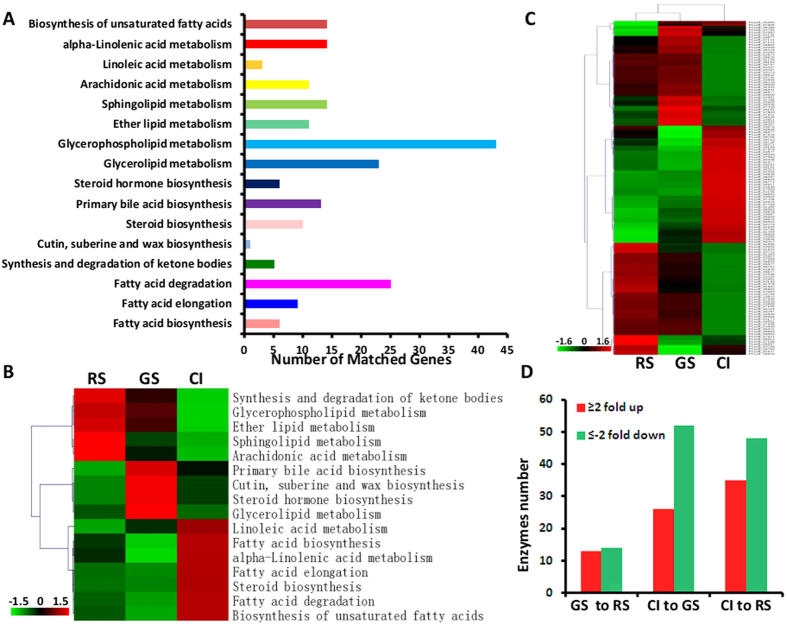
KEGG categories and gene expression of genes involved in lipid metabolism. (**A**) KEGG categories of *P. brassicae* lipid metabolism genes. (**B**) The expression levels of genes involved in lipid metabolism during different developmental stages. The levels were measured using the geometric mean of the TPM values of all genes in the corresponding group. To calculate the geometric mean of the TPM values, all TPM values of 0 were replaced by 0.001. (**C**) Gene expression in KEGG categories related to lipid metabolism. A colored bar indicating the normalized reads per LOG2 (RPKM) accompanies the expression profile. (**D**) A histogram showing enzyme number and changes in expression during the three life stages.

On the basis of genome analysis of *P. brassicae* from the single spore isolate ZJ-1 (Bi *et al*., submitted manuscript), eggNOG Functional Category Analysis defined 3534 genes. Among the 26 eggNOG subclasses, 133 genes matched with lipid transport and metabolism (Table S7). KEGG pathway analysis defined 3390 genes, which were distributed in 39 KEGG subclasses. 167 genes are involved in lipid metabolism (Table S8), which can be divided into 16 categories, with glycerophospholipid metabolism being the largest category (Fig. 6A). Fatty acid biosynthesis, fatty acid elongation and fatty acid degradation were highly abundant in the CI sample, indicating a high conversion of lipids in this active life-stage (Fig. 6B). Arachidonic acid metabolism was highly abundant in the resting spore stages (Fig. 6B). 162 candidated enzymes are involved in the lipid metabolism pathways (Table S9). Transcriptome analysis of genes involved in lipid metabolism showed that dynamic expressions in different development stages of *P. brassicae* (Fig. 6C and Table S10).

We observed a little change from the resting spores and germinating resting spores. 13 enzymes related genes were up-regulated, and 14 enzymes related genes were down-regulated (|Fold change| ≥2 and adjusted p-value <0.05). A drastic change was occurred in CI sample to RS or GS sample. From the CI sample to GS sample, 26 enzymes were up-regulated, and 52 enzymes were down-regulated. From the CI sample to RS sample, 35 enzymes were up-regulated, and 48 enzymes were down-regulated (Fig. 6D and Table S9). Lipid metabolism and lipid accumulation may enrich in cortical infection stage of gall development and spore formation.

## Discussion

Clubroot is a disease characterized by the formation of club-shaped galls on the roots of the infected plants. Root galls quickly disintegrate, release millions of resting spores into the soil, which can survive for years. Hence, lipid metabolism and lipid accumulation are critical for *P. brassicae* survival. Plasmodiophorids contains high lipid content and lipid droplets^12^,^13^. Lipid droplets have been found in resting spores and secondary plasmodia by TEM in *P. brassicae* (Fig. 1A–C). Nile red staining showed the development of *P. brassicae* zoosporic stages, including germinating spores, secondary zoospores, secondary plasmdia and resting spores (Fig. 1D–I). *P. brassicae* is an obligate biotroph protist, and is in need of nutrient supply from the host. In Schwelm *et al*. it is suggested that lipid precursors are being taken in from the host and then accumulated in the plasmodia and spores^7^. Nile red staining showed that lipid maybe not intake from the host, but the built up of lipid droplets in the plasmodia and their storage in the resting spores. In addition, Rolfe *et al*. show many genes associated with lipid metabolic processes were enriched in purified plasmodia stages^8^. We described the detail transcripts involved in lipid metabolism (Fig. 6), it has been speculated that lipid metabolism or accumulation faster in cortical infection stage than other development stages of *P. brassicae*.

Proteomics analysis revealed abundance of proteins on the lipid droplet surface, including enzymes, transport proteins, cell division-related proteins, transcription/translation proteins, ribosomal proteins, and many other unknown proteins (Fig. 3, Table S1). This diversity of lipid droplets-associated protein was also observed in earlier reported proteomics studies of multiple species, including oleaginous bacterium^24^, green algae^35^, *Drosophila*^27^,^28^, *C. elegans*^25^, adult mice^19^ and mammalian cells^26^. The number of enzymes present in lipid droplets is greater in mice than in *P. brassicae*^19^, which is greater in *P. brassicae* than that of the oleaginous bacterium PD630^24^. The structural lipid droplets-proteins, the PAT family of PLINs, are expressed in eukaryotes from humans to *Drosophila* as the marker lipid droplets-proteins^36^. However, there are no genes for apparent PLIN homologs in the *P. brassicae* genome. This result is similar to *C. elegans*^33^, raising the question whether *P. brassicae, C. elegans* and higher eukaryotic lipid droplets share conserved proteins and mechanisms of regulation. The proteome of *Drosophila* embryonic lipid droplets contained a large fraction of the total embryonic H2A, H2B, and H2A variant but not of H3 histone^27^. Our findings show that besides H2A and H2B, there are H3 and H4 histone existing in the proteome of *P. brassicae* lipid droplets. We do not understand whether H3 and H4 histone existing in lipid droplets was just by chance or by meaning. 14-3-3 protein plays a major role not only in *Paracoccidiodes brasiliensis* virulence and invasive infection, but also in the morphological program of this fungus^37^. Its role in the biology of the *P. brassicae* is unknown. Our next step may be to identify the specific marker proteins of lipid droplets in *P. brassicae*. Functional analysis of the gene associated with *P. brassicae* lipid droplets will be strongly shaped by its obligate biotrophic nature, while it is hard to investigate the function of some special lipid droplets-associated proteins in *P. brassicae*. In addition, it is apparent that lipid droplets are closely connected with many other cellular compartments (Table S6). It is possible that many of these contacts result in the transfer of lipids between compartments, and those droplets serve as the source of lipids for membrane expansion, energy production and signaling in *P. brassicae*.

Fatty acids can be released by lipases that gradually hydrolyze TAGs on the surface of lipid droplets. When cells are carbon/energy deprived, fatty acids are mobilized from stored neutral lipids^38^. Whole-cell fatty acid measurements showed that fatty acids C16:0, C18:1 ω9, C18:1 ω7 and C20:4 are present in resting spores of *P. brassicae*; and ARA (C20:4) is the single most abundant fatty acid, constituting 36% of the total amount of fatty acids^14^. Using GC-MS analysis, we identified two saturated fatty acids (C16:0 and C18:0) and five unsaturated fatty acids (C18:1Δ9, C18:1Δ11, C18:2, C20:4 and C20:5) from the lipid droplets (Fig. 4A, Table 1). It is interesting that C20:4 and C20:5 are rich in lipid droplets. Our results are not exactly the same with the results of previous studies, which might be due to the different methods and sources for sample separation. The same result is that C20:4 is an abundant fatty acid in the resting spores of *P. brassicae* and lipid droplets. KEGG pathway analysis showed that arachidonic acid metabolism did exist in the *P. brassicae* genome (Fig. 6A)^7^, and transcriptomes showed this metabolism was highly abundant in the resting spore stages (Fig. 6B)^7^. The rich unsaturated fatty acids in the resting spores of *P. brassicae* may help the pathogen to act against the unfavorable factors in the host cells and soil. *Pythium, Phytophthora* and *Halophytophthora* have been reported to produce EPA and ARA^39^,^40^. It is necessary to attempt to produce C20 unsaturated fatty acids from the clubroot galls, which might open new areas in *P. brassicae*.

Lipids are fundamental to all biological systems, and serve as an important fuel source. We believe that the *P. brassicae* transcriptome and lipid droplet proteome presented herein provide a starting point for understanding protist lipid droplets.

## Methods

### Plant Growth and *P. brassicae* inoculation

*Arabidopsis thaliana* (Col-0) and rapeseed (Hua shuang 4) were grown in a plant growth chamber maintained at 23 °C with a 16/8 h day/night cycle. Two-week-old seedlings were inoculated with 1 mL resting spore of *P. brassicae* strain ZJ-1 (1 × 10^7^ spores/mL). *P. brassicae* strain ZJ-1 was originally isolated from a diseased plant in a rapeseed field in Zhijiang city, Hubei Province, P R China. The virulence of single-spore isolated from *P. brassicae* strain ZJ-1 was tested on the differential hosts of Williams, showing the single-spore isolate derived from race 1^41^,^42^,^43^. The resting spores were extracted and purified according to the method described by T. Asano^44^. We purified the resting spores under 50% (w/v) sucrose solution by centrifugation and further purified by using continuous gradient of HS-40 colloidal silica (LUDOX, 40 wt% suspension in water; Sigma-Aldrich) as the protocol^45^. Resting spores were suspended in freshly prepared 2% chloramine-T solution (wt/vol) at room temperature for 20 min and then washed twice in sterile water by centrifugation at 1,700 g for 5 min. The resulting pellets were suspended in an antibiotic solution containing 1,000 ppm of colistin sulfate, 1,000 ppm of vancomycin hydrochloride and 6,000 ppm of cefotaxime sodium in distilled water and incubated at 25 °C in the dark. After 1 day, the suspension was washed twice in sterile water by centrifugation. The surface disinfested resting spores used immediately for extraction lipid droplets, and resting spores stored at 4 °C for inoculation the plants.

### Microscopic analysis

Light, fluorescent and TEM were carried out according to the following protocols. The main root nearest the surface was analyzed. For light microscopy, the roots were sectioned transversely, fixed in FAA buffer, washed, dehydrated, and embedded in paraffin. Then, the slides were stained with toluidine blue and observed under a Nikon light microscope. For fluorescent microscopy, the plant roots or resting spores were treated with nile red, which emits fluorescence over a broad range of wavelengths. The specimens were observed with a filter set designed for B excitation (i.e., FITC, Cy2, Alexa488, or GFP), which yielded the best images^46^. For electron microscopy, the roots were fixed in 2.5% glutaraldehyde for 4 h, and then post-fixed in 1% osmium tetroxide for 3 h. The samples were washed, dehydrated through an ethanol series, and embedded in London resin white. Ultrathin sections were examined by TEM (HITACHI, H-7000). Isolated lipid droplets were subjected to negative staining and examined by TEM.

### Nile red staining

Nile red (9-diethylamino-5H-benzo (alpha) phenoxazine-5-one) (Sigma–Aldrich) was prepared as a stock solution at a concentration of 250 mg/L in acetone. Resting spores or plant roots were incubated with nile red solution (10 μg/mL nile red in acetone) for 3–5 s; for fluorescence microscopy, we removed the excess dye by briefly rinsing in PBS or ddH_2_O^46^.

### Lipid droplets isolation

A reported method was modified and used to isolate lipid droplets from multiple species^47^. Surface disinfested resting spores were harvested by centrifugation at 4,000 g for 10 min in a 50 mL tube, and the spores were washed twice in 30 ml of buffer A (20 mM tricine and 250 mM sucrose, pH 7.8). The samples were suspended in buffer A plus 0.2 mM PMSF and kept on ice for 20 min. The cells were then lysed by plunging three times in a French press at 1,500 bar at 4 °C. The homogenate was centrifuged at 3,000 × *g* for 10 min at 4 °C to remove nuclei, cell debris and unbroken cells to yield a supernatant containing the postnuclear fraction. The supernatant (8 mL) was loaded into several SW40 tubes containing 2 ml buffer B (20 mM HEPES, 100 mM KCl, 2 mM MgCl_2_, pH 7.4) and then was centrifuged at 10,600 × *g* for 1 h at 4 °C (Beckman SW40). The most important physical property of lipid droplets is their low density, and thus they float on the top of all aqueous gradients after centrifugation. The lipid droplets fraction at the top of the sucrose gradient was collected using a pipette tip.

### Mass spectrometry (MS) analysis

Lipid droplet proteins were separated on a 10% SDS-PAGE gel. The bands of interest from the sample were cut from SDS-PAGE gels. In-gel digestion of each slice was performed as follows. Each slice was successively destained with 30% ACN/100 mM NH4HCO3, and then dehydrated with 100% acetonitrile. Proteins were reduced with 100 mM DTT at 56 °C for 30 min and alkylated by 200 mM IAA in the dark at room temperature for 20 min. Finally, gel pieces were thoroughly washed with 100% ACN and completely dried in a SpeedVac. Proteins were incubated for 30 min in 2.5–10 ng/μL Trypsin on ice and leaving for 20 h at 37 °C. The gel pieces were extracted twice with 60% ACN/0.1% TFA, and then sonicated for 15 min. All liquid samples from the three extractions were combined and dried in a SpeedVac. Dried peptide samples were dissolved in 60 μL 0.1% FA. Samples were loaded onto a C18 trap column with an auto-sampler and then eluted onto a C18 column (100 mm × 100 μm) packed with Sunchrom packing material (SP-120-3-ODS-A, 3 mm) for nano-LC-ESI-LTQ MS/MS analysis. All the MS/MS data were searched against our assembled and annotated genome sequence by the Mascot 2.2 program. Only proteins that matched at least two unique peptides were accepted for identification.

### GC-MS analysis

The purified lipid droplets fractions were extracted with ether and petroleum ether (1:1, v/v), and the lower phase of the extract was collected. The total lipid was methyl esterified in 0.4 M KOH/Methanol for 1 hour. After the addition of ddH_2_O, the samples were centrifuged and the supernatant was collected. The fatty acid methyl ester was subjected to GC-MS using a Shimadzu gas chromatograph equipped with a quadrupole mass spectrometer for electron impact ionization (GC-MS-QP2010). A SH Stabilwax DA column (30 m length, 0.25 mm diameter, and 0.25 μm film thickness) was used to separate the fatty acid methyl ester at a flow rate of 1.0 mL/min. The injector temperature was set to 200 °C and the transfer line temperature to 280 °C. The GC oven was programmed as follows: after 2 min at 50 °C, the temperature was increased at 30 °C/min to 150 °C, then at 15 °C/min to 230 °C. The total run duration was 25 min. GC/MS analysis was conducted in the full scan mode (*m*/*z* 35–600). Qualitative analysis was based on the characteristic ions of the fatty acid methyl esters and their relative retention times. Content qualitative analysis was based on the peak areas of the fatty acids.

### Measurement of TAG levels

Samples of unicellular organisms such as *Saccharomyces cerevisiae, Escherichia coli, Pseudomonas syringae, Chlamydomonas reinhardtii* and conidia of *Trichoderma viride* were cultured in appropriate culture conditions. Resting spores of *P. brassicae* were extracted from galls of rapeseed and then surface disinfested. The sample suspensions were harvested by centrifugation at 4,000 *g* for 10 min in a 50-ml tube, washed twice with 10 ml PBS, and lysed by compressing three times with a French press at 1,500 bar at 4 °C. Whole sample lysates were then centrifuged at 12,000 *g* for 5 min at 4 °C. The lysates were examined under a microscope to ensure that the cells were completely broken. The TAG content of the supernatant was measured using an E1003 triglyceride assay kit (Applygen Technologies, China), and the protein concentration was quantified using a Pierce BCA Protein Assay Kit (Thermo, USA).

### TLC

*P. brassicae* resting spores were extracted twice with a mixture of chloroform, methanol, and medium (1:1:1, v/v/v). Purified lipids were extracted in a chloroform and acetone mixture (1:1, v/v). Organic phases were collected and dried under high-purity nitrogen gas. Total lipids were dissolved in 100 μL chloroform, vortexed, and centrifuged for 1 min at 10,000 rpm. The samples were then subjected to TLC analysis with Whatman Purasil TM 60FÅ silica gel plates (Merck; Germany). The plates were developed in a hexane, diethyl ether and acetic acid (80:20:1, v/v/v) solvent system to separate neutral lipids and were visualized using iodine vapor and UV light.

### RNA preparation, extraction, sequencing and transcriptome analysis

*P. brassicae* RNA from samples was generated from resting spores (RS), germinating spores (GS) and secondary plasmodia (cortical infection, CI). Resting spores were extracted from clubroot using above-mentioned methods. Resting spores were treated with rapeseed root exudate solution for 24 hours germination, and then were inoculated to sterile rapeseed seedlings in the sterile plates at 22 °C for 48 hours in darkness. Roots were extensively washed in sterile water and removed, and primary zoospores were collected in the remaining water and solution and determined under microscope. As plasmodia are difficult to be isolated from the infected rapeseed roots, we collected the galls infected by *P. brassicae* for 21 days, and the plasmodia were determined under microscope. RNA was extracted by using Trizol Reagent (Takara, Dalian, China) following the standard protocol. After RNA amplification, libraries were constructed for sequencing on an Illumina NextSeq 500 platform for paired-end 2 × 150 bp system and sequenced at the Shanghai Personal Biotechnology Company Limited (Shanghai, China) according to the manufacturer’s specifications. The RNA-Seq raw reads were processed to obtain high quality reads by removing the adapter sequences and low quality bases at the 3′ end, trimming low-quality bases (Q < 20) from the 3′ to 5′ ends of the remaining reads. Reads filtering out reads containing ‘N’, reads greater than 50 bp were considered for analysis. The filtered reads were mapped to the *P. brassicae* genome using Tophat v2.0.9 (http://tophat.cbcb.umd.edu/)^48^, and analyzed using with HTSeq^49^ and DESeq^50^. To comparing the expression pattern of each gene between samples, the abundance of each transcript was normalized by RPKM^51^. The heat map of the clustered genes and samples was generated by the clustering affinity search technique (CAST) assay through MultiExperiment Viewer v4.9 software packag^52^. Fold changes (log_2_Ratio) were estimated according to the normalized gene expression level in each sample. The mapped genes with a threshold of p-value ≤0.05 and the absolute value of log_2_Ratio ≥1 between different samples were identified as significantly differential genes.

### Data access

This Whole Genome Shotgun project has been deposited at DDBJ/ENA/GenBank under the accession MCBL00000000. The version described in this paper is version MCBL01000000. The expression data sets used in this study are available at the NCBI Gene Expression Omnibus (GEO) (http://www.ncbi.nlm.nih.gov/geo/) under accession number SRP079943.

## Additional Information

**How to cite this article**: Bi, K. *et al*. Integrated omics study of lipid droplets from *Plasmodiophora brassicae. Sci. Rep.* **6**, 36965; doi: 10.1038/srep36965 (2016).

**Publisher's note**: Springer Nature remains neutral with regard to jurisdictional claims in published maps and institutional affiliations.

## Supplementary Material

Supplementary FigureS1–3

Supplementary Table S1–10

## Figures and Tables

**Table 1:** The free fatty acid composition of lipid droplets of *P. brassicae* resting spores determined by GC-MS.

Number	Fatty acid	Retention time	Peak area %
1	C16:0	12.583	36.4
2	C18:0	14.146	40.2
3	C18:1Δ9	14.352	6.5
4	C18:1Δ11	14.413	1
5	C18:2Δ9, 12	14.817	1.7
6	C20:4Δ5, 8, 11, 14	18.289	10
7	C20:5Δ5, 8, 11, 14, 17	19.592	4.2

The quantitative analysis was based on the peak areas of the fatty acids.
