# Supplementary material for: Integrated omics study of lipid droplets from Plasmodiophora brassicae
Source: Sci Rep. 2016 Nov 22;6:36965. doi: 10.1038/srep36965 (PMC5118790; doi:10.1038/srep36965)
Supplement: Supplementary FigureS1–3 [file srep36965-s1.zip › supplementary FigureS1-3.pptx]

## Slide 1
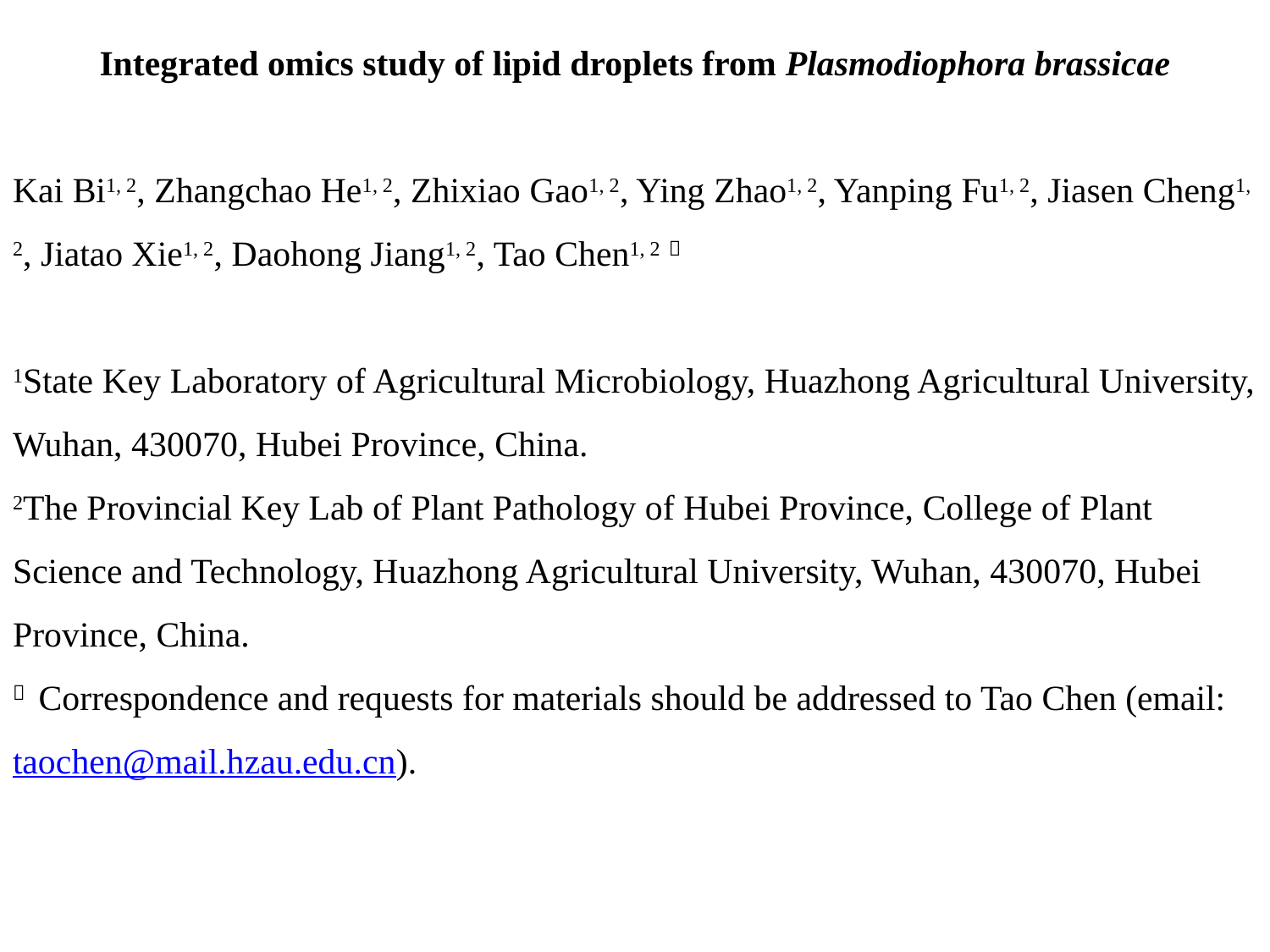

Integrated omics study of lipid droplets from Plasmodiophora brassicae
Kai Bi1, 2, Zhangchao He1, 2, Zhixiao Gao1, 2, Ying Zhao1, 2, Yanping Fu1, 2, Jiasen Cheng1, 2, Jiatao Xie1, 2, Daohong Jiang1, 2, Tao Chen1, 2＊
1State Key Laboratory of Agricultural Microbiology, Huazhong Agricultural University, Wuhan, 430070, Hubei Province, China.
2The Provincial Key Lab of Plant Pathology of Hubei Province, College of Plant Science and Technology, Huazhong Agricultural University, Wuhan, 430070, Hubei Province, China.
＊ Correspondence and requests for materials should be addressed to Tao Chen (email: taochen@mail.hzau.edu.cn).

## Slide 2
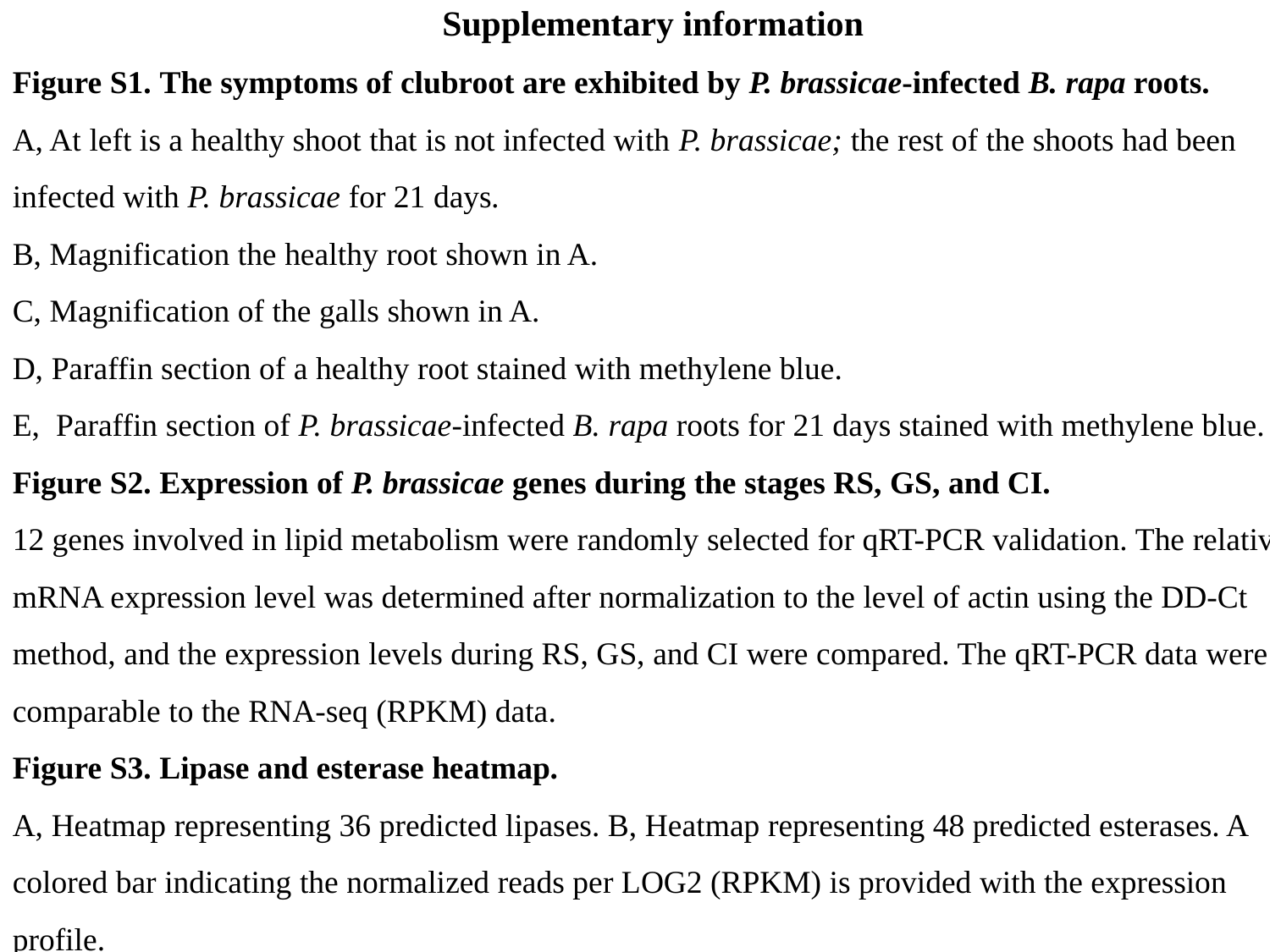

Supplementary information
Figure S1. The symptoms of clubroot are exhibited by P. brassicae-infected B. rapa roots.
A, At left is a healthy shoot that is not infected with P. brassicae; the rest of the shoots had been infected with P. brassicae for 21 days.
B, Magnification the healthy root shown in A.
C, Magnification of the galls shown in A.
D, Paraffin section of a healthy root stained with methylene blue.
E, Paraffin section of P. brassicae-infected B. rapa roots for 21 days stained with methylene blue.
Figure S2. Expression of P. brassicae genes during the stages RS, GS, and CI.
12 genes involved in lipid metabolism were randomly selected for qRT-PCR validation. The relative mRNA expression level was determined after normalization to the level of actin using the DD-Ct method, and the expression levels during RS, GS, and CI were compared. The qRT-PCR data were comparable to the RNA-seq (RPKM) data.
Figure S3. Lipase and esterase heatmap.
A, Heatmap representing 36 predicted lipases. B, Heatmap representing 48 predicted esterases. A colored bar indicating the normalized reads per LOG2 (RPKM) is provided with the expression profile.

## Slide 3
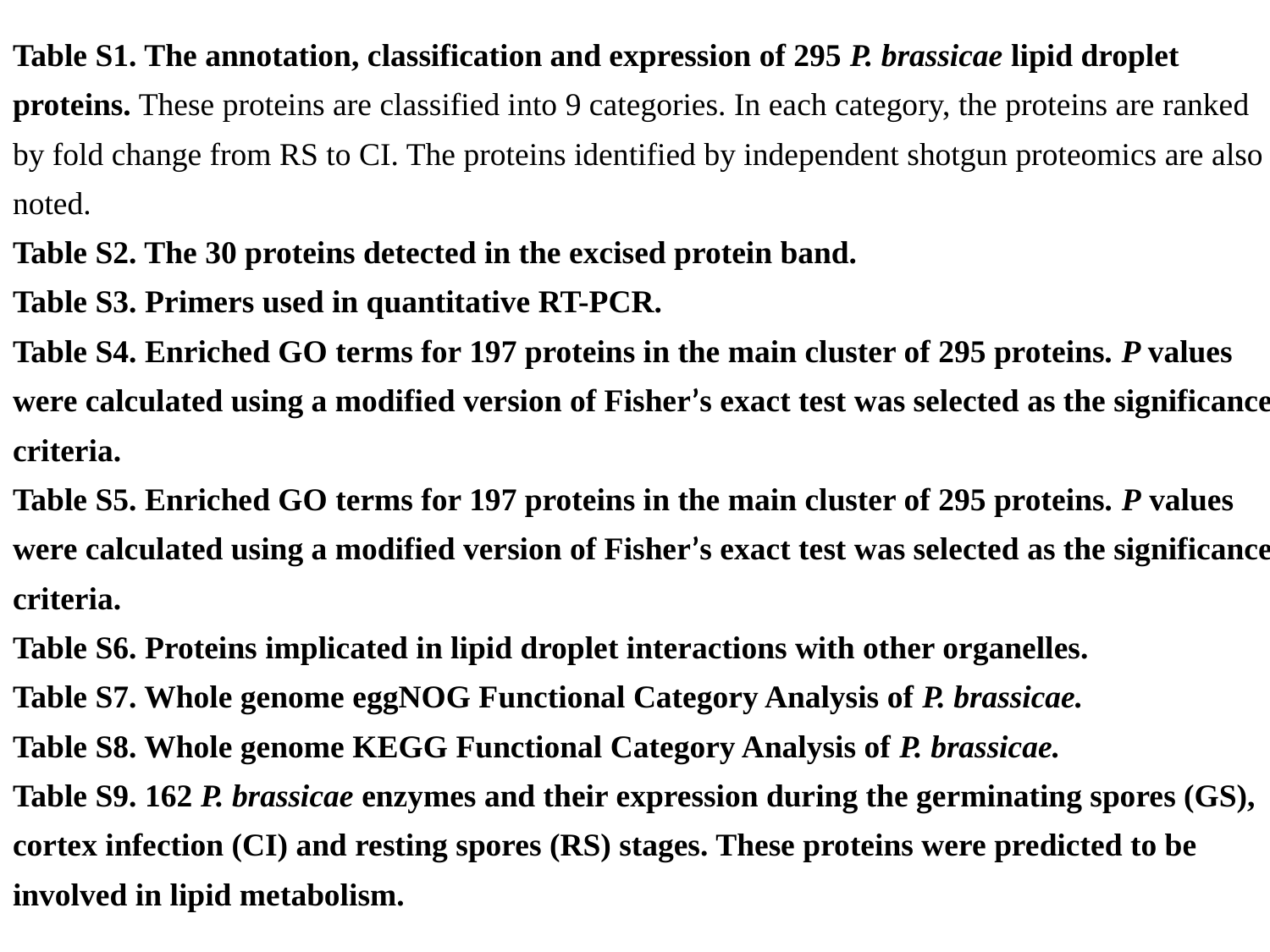

Table S1. The annotation, classification and expression of 295 P. brassicae lipid droplet proteins. These proteins are classified into 9 categories. In each category, the proteins are ranked by fold change from RS to CI. The proteins identified by independent shotgun proteomics are also noted.
Table S2. The 30 proteins detected in the excised protein band.
Table S3. Primers used in quantitative RT-PCR.
Table S4. Enriched GO terms for 197 proteins in the main cluster of 295 proteins. P values were calculated using a modified version of Fisher’s exact test was selected as the significance criteria.
Table S5. Enriched GO terms for 197 proteins in the main cluster of 295 proteins. P values were calculated using a modified version of Fisher’s exact test was selected as the significance criteria.
Table S6. Proteins implicated in lipid droplet interactions with other organelles.
Table S7. Whole genome eggNOG Functional Category Analysis of P. brassicae.
Table S8. Whole genome KEGG Functional Category Analysis of P. brassicae.
Table S9. 162 P. brassicae enzymes and their expression during the germinating spores (GS), cortex infection (CI) and resting spores (RS) stages. These proteins were predicted to be involved in lipid metabolism.

## Slide 4
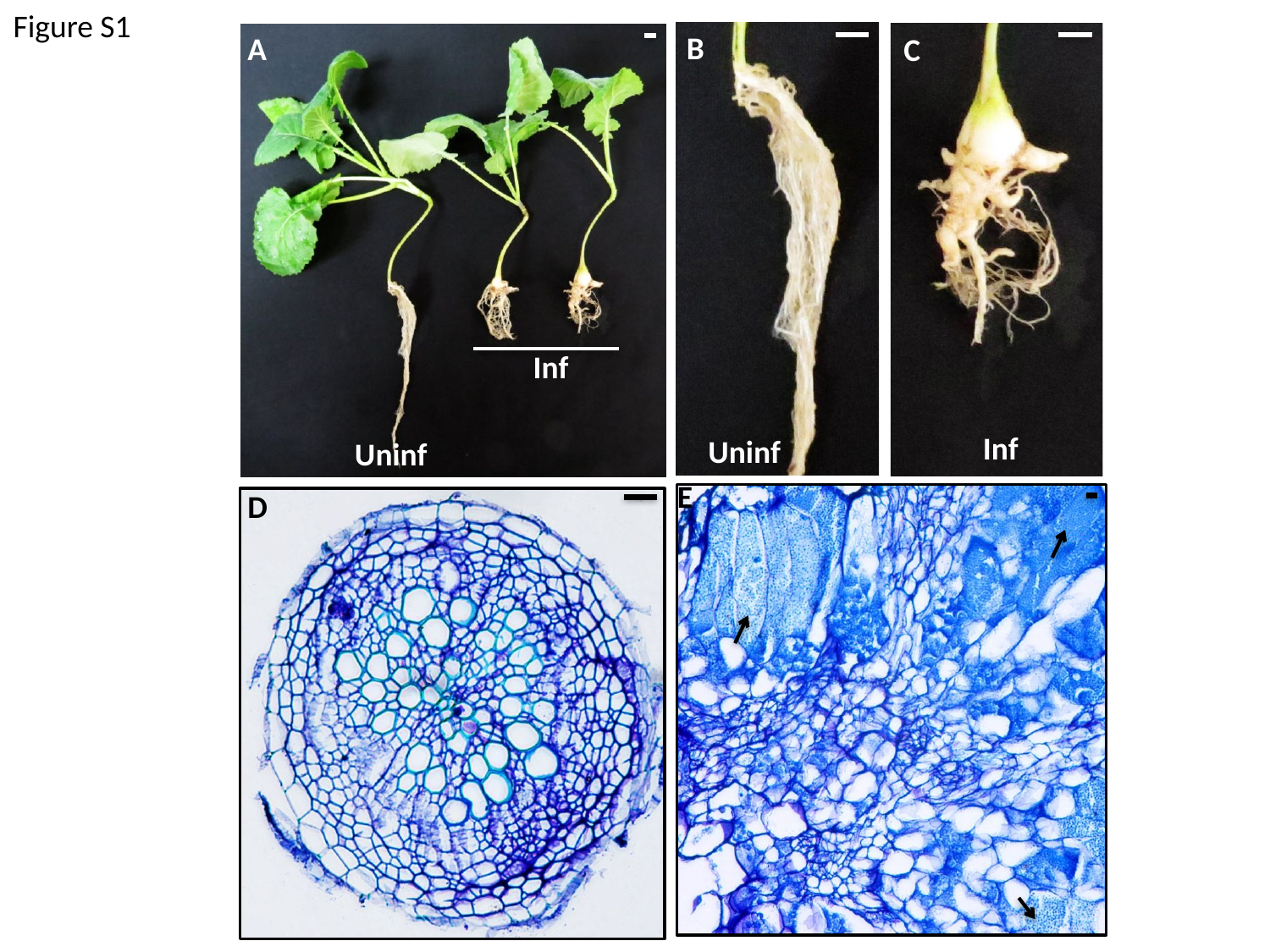

Figure S1
B
A
C
Inf
Inf
Uninf
Uninf
E
D

## Slide 5
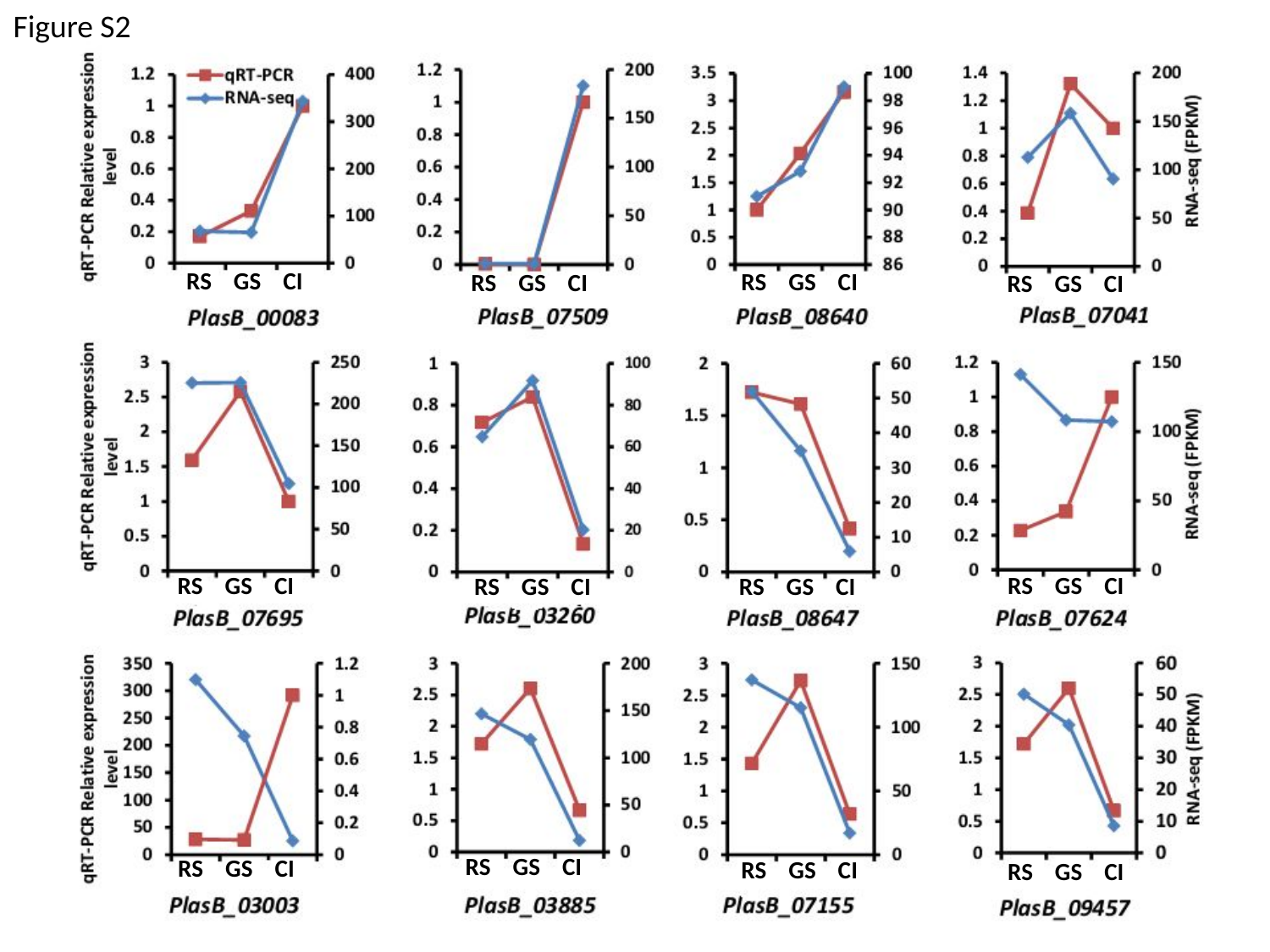

Figure S2
RS GS CI
RS GS CI
RS GS CI
RS GS CI
RS GS CI
RS GS CI
RS GS CI
RS GS CI
RS GS CI
RS GS CI
RS GS CI
RS GS CI

## Slide 6
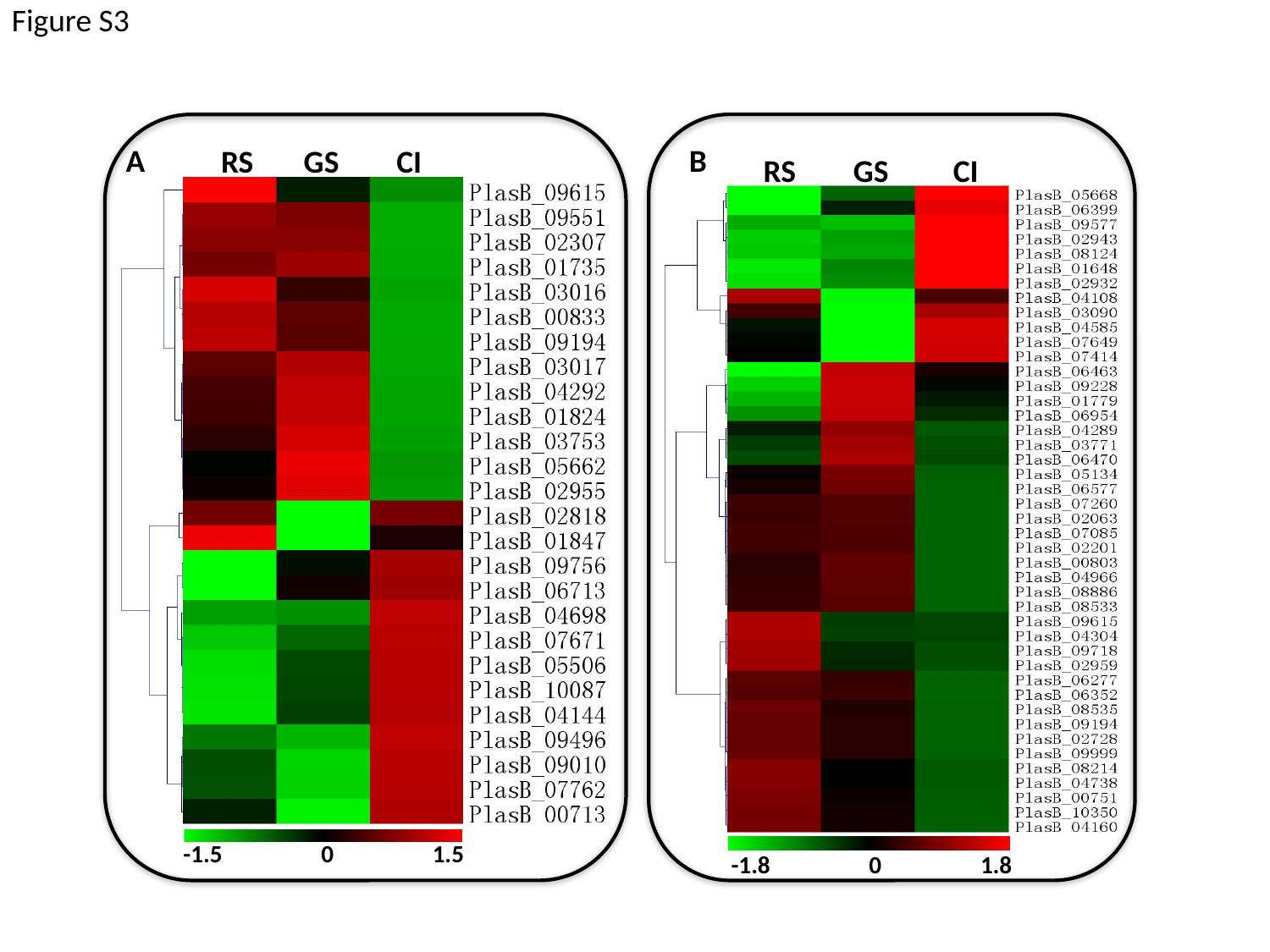

Figure S3
A
B
RS GS CI
RS GS CI
-1.5 0 1.5
-1.8 0 1.8
